# Evaluating disease burden in chronic kidney disease screening using a micro-simulation model

**DOI:** 10.3389/fpubh.2025.1608445

**Published:** 2025-09-12

**Authors:** Yang Li, Yuqin Ma, Pei Liu, Ping Xu, Guangfeng Duan

**Affiliations:** ^1^Department of Health Service Management, Faculty of Health Service, Naval Medical University, Shanghai, China; ^2^Department of Mathematics and Physics, Naval Medical University, Shanghai, China

**Keywords:** micro-simulation, screening interventions, disease burden, chronic renal insufficiency, cost of illness

## Abstract

**Introduction:**

Chronic kidney disease (CKD) has a high prevalence, poor prognosis, and high medical costs, and awareness of the disease is low. Therefore, in this study, we aimed to simulate and analyze the evolution of CKD burden among different groups at high risk of CKD in Shanghai, with or without screening intervention, and provide a quantifiable basis for the selection of screening intervention strategies for CKD.

**Methods:**

A micro-simulation model was constructed to analyze the evolution of CKD burden using data from CKD screening of the population in the Jing’an and Minhang Districts of Shanghai, China, from January 2015 to December 2020. SAS Statistical Software 9.4 was used to simulate and analyze the evolution of disease burden under different screening intervention strategies.

**Results:**

By 2033, screening interventions for high-risk groups with hypertension, diabetes, and an age of 65 years and older would be associated with 6,250 fewer patients with end-stage renal disease. Furthermore, the number of patients with end-stage renal disease would be reduced to only 41.64% of the projected number of patients without screening intervention, leading to a general improvement in the quality of life of the population, better quality-adjusted life-years, and a reduction in the economic burden of disease.

**Discussion:**

The results of this study highlight the importance of combining the concepts of integrated prevention and treatment of chronic diseases to improve screening and intervention of CKD for people with hypertension, diabetes, and those aged 65 years and older, thereby effectively reducing the number of patients with end-stage renal disease, lowering the cost of treatment and intervention, and improving the quality of life of the population.

## 1 Introduction

Chronic kidney disease (CKD) refers to structural or functional impairments in the kidneys caused by various factors. Due to the high prevalence, poor prognosis, high medical costs, and low awareness of the disease, CKD has become a serious threat to human health, following cardiovascular diseases, diabetes, and malignant tumors (1–7). According to relevant statistics, the global prevalence of CKD is 10.1–13.3%. It is projected that by 2040, CKD will become the fifth leading cause of mortality worldwide (8). In China, a cross-sectional epidemiological study conducted in 2012 showed a CKD prevalence of 10.8% among individuals aged 18 years and older (9). In 2015, Shanghai included the prevention and control of CKD in its 3-year public health action plan. The Jing’an and Minhang districts were selected as screening bases for high-risk groups with CKD, the high-risk population for CKD refers to individuals who have at least one of the following risk factors: ① older adult(s) aged 65 and above; ② Hypertension; ③ Diabetes; ④ Hyperuricemia; ⑤ Has a family history of kidney disease, and the results revealed a detection rate of 25.75%. Specifically, the detection rates were 23.50% for males and 27.29% for females (10, 11). CKD often has a subtle onset; however, most patients only seek medical attention when they have symptoms, which typically occur in the middle to late stages of the disease. When CKD progresses to the end stage, it poses a serious threat to patients’ lives.

To alleviate the heavy social burden caused by CKD and the considerable medical expenses associated with dialysis, health system needs to prioritize measures to better manage patients with early-stage CKD. Although there is consensus on the early prevention and treatment of CKD (11), the practical evaluation of prevention and treatment strategies in real society often faces many constraints. This is mainly manifested in the difficulty of evaluating the effectiveness of the early prevention and treatment of CKD. CKD has a long course of development and often requires years of follow-up, making it difficult to obtain a health economic evaluation of the impact of changes in disease burden on the population in the presence or absence of intervention through prevention and control strategies in real society.

Furthermore, the traditional “intervention control” method for evaluating prevention and control strategies requires high implementation conditions and is difficult to implement. For example, problems such as high resource consumption, lengthy cycles, and a complex intervention and comparison policy environment during evaluation implementation are encountered. Moreover, the comparison of disease treatment involves ethical issues that are difficult to address. During the research process, there are many human interventions that research participants would not easily accept, resulting in a limited quantity and quality of evidence.

Therefore, in this study, we aimed to construct a micro-simulation model for the evolution of CKD in patients. The micro-level data files, containing demographic information and CKD prevalence, were integrated to simulate changes in individual disease occurrence and progression due to different screening intervention strategies for CKD. Based on this, the progression of disease burden in the population was analyzed and recommendations were proposed for the selection of prevention and treatment strategies.

## 2 Materials and methods

### 2.1 Data sources

#### 2.1.1 Literature collection

The probability of disease state transition and utility data related to disease burden were collected through systematic literature searches. We primarily analyzed disease state transitions, with or without screening and intervention, which were mainly obtained from epidemiological surveys. Probability parameters for disease state transitions in CKD with screening intervention were obtained with a focus on relevant intervention studies. Studies have shown that angiotensin-converting enzyme inhibitors (ACEIs) and angiotensin II receptor blockers (ARBs) reduce urinary protein levels. The relative effectiveness of ACEIs and ARBs compared with a placebo or no treatment was obtained from the latest relevant meta-analyzes. The literature search databases included MEDLINE, the Cochrane Library, and EMBASE. The model parameters retrieved through the search terms are shown in Table 1.

**Table 1:** Parameter retrieval plan for the disease burden evolution model of chronic kidney disease.

Category	Keyword
Health status and outcome events	Hemodialysis, HD; End-stage renal disease, ESRD; Chronic kidney disease, CKD; Microalbuminuria; Hypertension; Albuminuria; Macroalbuminuria; Proteinuria; Diabetes.
Diagnosis and intervention	Screen; Angiotensin receptor blocker, ARB; Angiotensin-converting enzyme inhibitors, ACEI; Renin–Angiotensin System Inhibitor, RAS Inhibitor; Incidence; Prevalence; Mortality.
Research process design	Randomized control trials, RCT; Meta-analysis, Epidemic investigation; Cost–Utility.

The collected indicators included the probability of death, incidence rate of end-stage renal disease, mortality of end-stage renal disease, mortality of diagnosed CKD, incidence rate of end-stage renal disease in diagnosed CKD, mortality of end-stage renal disease in diagnosed CKD, mortality of undetected CKD, incidence rate of undetected CKD, mortality of CKD, morbidity of end-stage renal disease of CKD, probability of death due to diagnosed and intervened CKD, probability of end-stage renal disease onset due to diagnosed and intervened CKD, and the probability of death due to diagnosed and intervened end-stage renal disease in CKD.

We calculated the probability of state transition based on the collected event occurrence rate.

#### 2.1.2 Survey data collection

The data in this study were gathered from a CKD screening population in the Jing’an and Minhang Districts of Shanghai, China, from January 1, 2015, to December 22, 2020. The collected information included demographic and sociological characteristics, height, weight, diastolic and systolic blood pressure, health insurance type, screening date, urinary protein concentration, urinary albumin–creatinine ratio, blood creatinine concentration, estimated glomerular filtration rate, and screening results. The total number of individuals screened was 104,593. Among them, 28,931 were known to have CKD before screening, and the remaining 75,662 individuals had no signs of CKD. The prevalence of CKD in the population and medical costs were derived from this database.

Data on the prevalence of CKD and its direct medical costs were collected from the CKD screening database.

### 2.2 Research methods

#### 2.2.1 Module for initial data file

Based on the changes in the registered population by age in Shanghai, this study carried out predictions until the beginning of 2023. The number of individuals in the population by age and disease stage in the starting year was estimated considering the distribution of high-risk factors (hypertension, diabetes) related to CKD and the prevalence of CKD among high-risk groups. Accordingly, microdata files were constructed to serve as the foundation for the simulation.

#### 2.2.2 Module for evaluating disease state

**Figure 1:**
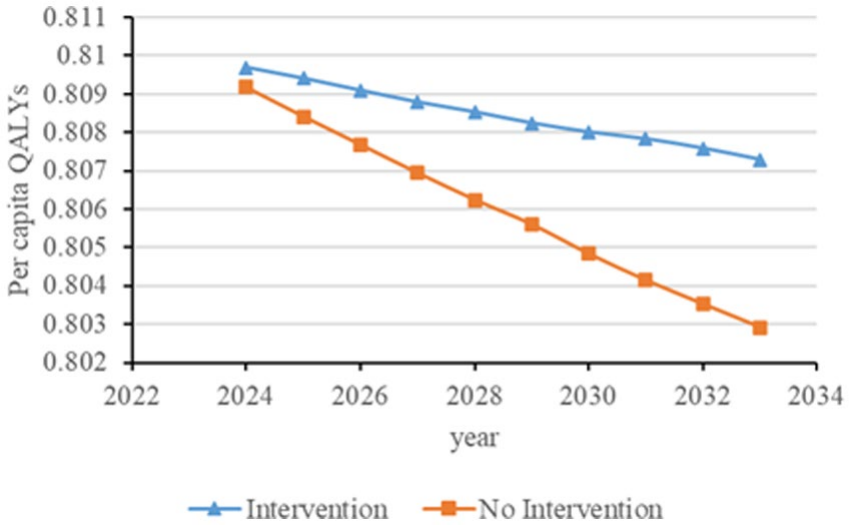
Schematic diagram of disease state transitions in chronic kidney disease.

Based on the natural progression of CKD combined with survey data and literature search results, we obtained the transition probabilities among different CKD states. The parameters were mainly derived from large-scale queue analysis and meta-analysis results, combined with sensitivity analysis results, to synchronize transition probabilities from multiple sources. The model simulated the annual progression of the disease for individual patients. Figure 1 illustrates the disease state transitions in CKD.

#### 2.2.3 Module for annual adjustment and result analysis

This module was used to make necessary adjustments and prepare data for the next year’s simulation. Subsequently, a comprehensive analysis of the simulation results was performed. The model simulated CKD-related mortality events in the population based on the mortality probabilities that were obtained for different CKD states. After the simulation, the model generated results, which were then comprehensively analyzed.

### 2.3 Data analysis

SAS statistical software 9.4 was used to simulate and analyze the evolution of disease burden under different screening intervention strategies.

## 3 Results

### 3.1 Construction of micro-simulation data files for CKD

Using data from CKD screening results and estimated new cases identified through screening, initial microdata files for individuals with CKD were constructed. The construction process was as follows:

1. It was assumed that the distribution of high-risk groups for CKD (hypertension and diabetes) in Shanghai’s registered population matched the distribution of high-risk groups participating in the screening. The age of the screened patients followed a normal data distribution, with a mean age of 71.45 years and a standard deviation of 8.18 years. Based on the proportion of the population with a high risk of CKD (10%), microdata with age characteristics for high-risk individuals with CKD in Shanghai’s registered population were randomly generated. In this paper, we considered the minimum age to be 45 years.
2. A new micro-database of the screened patients with CKD was randomly generated for age and CKD stage characteristics. The screening data for patients with CKD, including the prevalence and proportion of newly identified patients, were combined with the generated microdata containing age and CKD stage characteristics. Table 2 presents the constructed microdata for newly screened patients with CKD in Shanghai by age and CKD stage.

**Table 2:** Microdata constructed for newly screened patients with CKD in Shanghai by age and CKD stage.

Age group (years)	Frequency	Percentage (%)	Effective percentage (%)	Cumulative percentage (%)
45.00	128	0.4	0.4	0.4
50.00	546	1.5	1.5	1.9
55.00	1876	5.3	5.3	7.2
60.00	4,546	12.8	12.8	20.0
65.00	7,343	20.7	20.7	40.8
70.00	8,474	23.9	23.9	64.7
75.00	6,719	19.0	19.0	83.7
80.00	3,761	10.6	10.6	94.3
85.00	1,510	4.3	4.3	98.6
90.00	417	1.2	1.2	99.7
95.00	79	0.2	0.2	100.0
100.00	13	0.0	0.0	100.0
Total	35,412	100.0	100.0	

CKD, chronic kidney disease.

### 3.2 Obtained model simulation parameters

#### 3.2.1 Disease state transition parameters

Transition probabilities or event occurrence probabilities are typically not directly available in the literature and are usually calculated from event incidence rates. Transition probabilities, or event occurrence probabilities, were calculated based on event incidence rates. Table 3 shows the query results.

**Table 3:** Query results for disease state transition probability parameters.

Items	Indicators	Range	Data sources
General population			
Mortality rate of general population	R	8.87‰	Shanghai Municipal Bureau of Statistics (22)
End-stage renal disease mortality rate	R	3.38%	Shanghai Dialysis Annual Registration Report
Before intervention			
CKD mortality rate	HR	1.63 (1.5–1.77)	Matsushita et al. (23)
End-stage renal disease incidence rate	RR	0.79 (0.70–0.90)	Deng et al. (24)
After intervention			
CKD mortality rate	HR	0.83 (0.78–0.87)	Qin et al. (25)
End-stage renal disease incidence rate	RR (8.89, 11.13%)	0.79 (0.70–0.90)	Deng et al. (24)

CKD, chronic kidney disease; HR, hazard ratio; RR, relative risk.

#### 3.2.2 Utility parameters

Health utility in the model was measured by quality-adjusted life years (QALYs), where perfect health had a QALY value of 1, and death had a QALY value of 0. Data on the disease state in CKD were obtained from relevant literature. An analysis in Japan showed that the QALY values for CKD stages 1–4 were approximately 0.81. The QALY value of 0.658 for end-stage renal disease was obtained from the Chronic Renal Insufficiency Cohort study.

#### 3.2.3 Medical cost data

Table 4 presents the outpatient medical expenditures for patients with CKD. The data indicate that outpatient medical expenses for patients with end-stage disease were significantly higher than those for patients with pre-end-stage disease. The discount rate for costs during the simulation process was set at 5%.

**Table 4:** Outpatient expenditure for individuals with CKD.

Stages	Number of patients	Average age (years)	Per capita consumption (yuan)
Stages I–IV	10,508	77	4,159
Stage V	214	71	107,209

CKD, chronic kidney disease.

CKD community screening mainly relies on urinalysis and blood creatinine tests for assessment. Therefore, the screening cost mainly comprises these two parts. Both costs were determined based on the Shanghai healthcare service prices, which were 4 and 8 yuan, respectively. The cost of treatment primarily includes ACEI and ARB medication expenses, which, based on market price research, amounts to 30 yuan per week.

### 3.3 Results of CKD burden estimation

By constructing the micro-simulation model, we focused on simulating the evolution of disease burden over the next 10 years for patients identified in the year 2023 under scenarios with and without screening and management interventions. A total of five situations were simulated: first, screening and intervention in the hypertension group; second, screening and intervention in the diabetes group; third, screening and intervention in both the hypertension and diabetes groups; fourth, screening and intervention for individuals aged 65 years and older; and fifth, screening and intervention for individuals with hypertension, diabetes, and aged 65 years and older. We analyzed the data of individuals aged 45 years and older.

Table 5 presents the evolution of CKD disease burden in the presence and absence of screening intervention. From the perspective of the change in the number of patients with end-stage disease each year, it is evident that the screening intervention significantly reduces this number in different screened groups compared with those without intervention. The growth rate of patients with end-stage renal disease each year is effectively controlled through screening interventions. Judging by the simulation results of 2024, without intervention, the increase in patients with end-stage renal disease in the older adult, hypertension, and diabetes groups roughly aligns with the reported annual increase of 2000 cases in Shanghai (12, 13), signifying the accuracy of the simulation results.

**Table 5:** Evolution of patients with end-stage renal disease under different screening intervention strategies.

Year	Hypertension		Diabetes		Hypertension and diabetes		Older adults		Older adults, hypertension and diabetes	
	Intervention	No intervention	Intervention	No intervention	Intervention	No intervention	Intervention	No intervention	Intervention	No intervention
2024	520	1,290	290	670	550	1,410	540	1,480	690	1860
2025	1,010	2,480	500	1,220	1,070	2,570	1,030	2,760	1,290	3,360
2026	1,490	3,620	770	1740	1,620	3,670	1,490	3,920	1930	4,810
2027	1920	4,520	920	2,180	1980	4,610	1870	4,900	2,400	6,000
2028	2,220	5,330	1,080	2,640	2,410	5,470	2,260	5,630	2,920	7,060
2029	2,530	6,030	1,230	3,170	2,760	6,350	2,620	6,650	3,330	8,160
2030	2,830	6,900	1,420	3,480	2,960	7,060	2,830	7,260	3,550	8,980
2031	3,050	7,430	1,570	3,780	3,150	7,590	3,060	7,800	3,810	9,640
2032	3,270	7,920	1740	4,080	3,400	8,080	3,230	8,240	4,150	10,270
2033	3,490	8,270	1810	4,400	3,640	8,440	3,480	8,650	4,460	10,710

**Figure 2:**
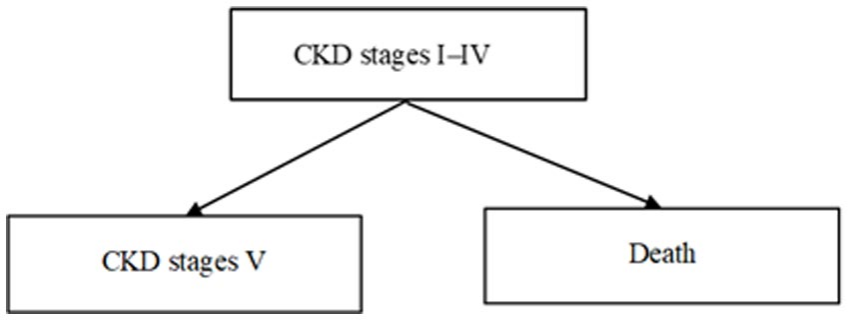
Changes in per capita quality-adjusted life-years before and after hypertension screening and intervention. QALYs, quality-adjusted life-years.

**Figure 3:**
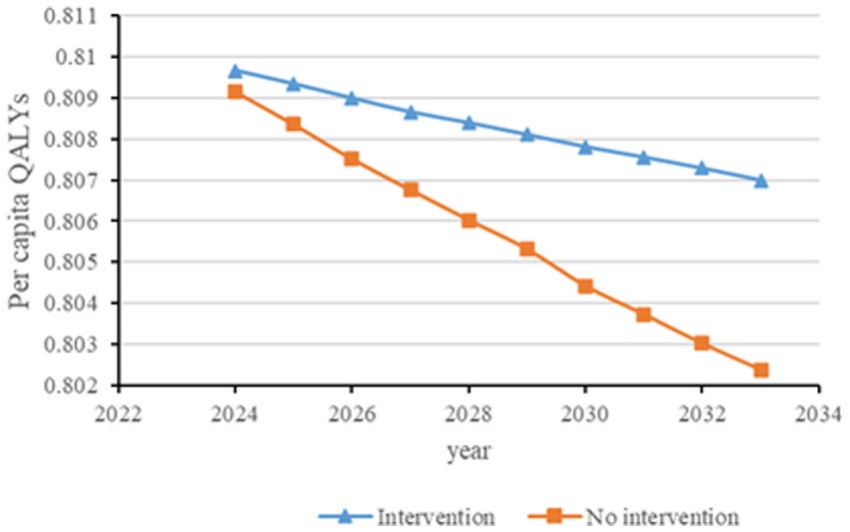
Changes in per capita quality-adjusted life-years before and after diabetes screening and intervention. QALYs, quality-adjusted life-years.

**Figure 4:**
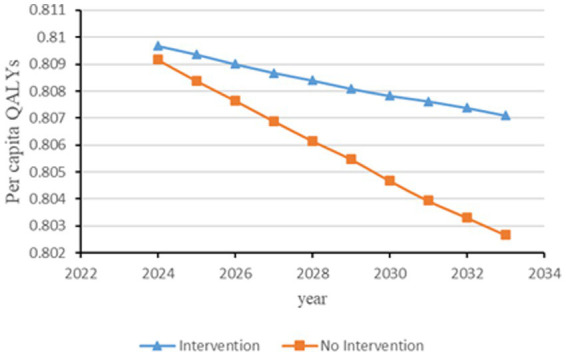
Changes in per capita quality-adjusted life-years before and after hypertension and diabetes screening and intervention. QALYs, quality-adjusted life-years.

**Figure 5:**
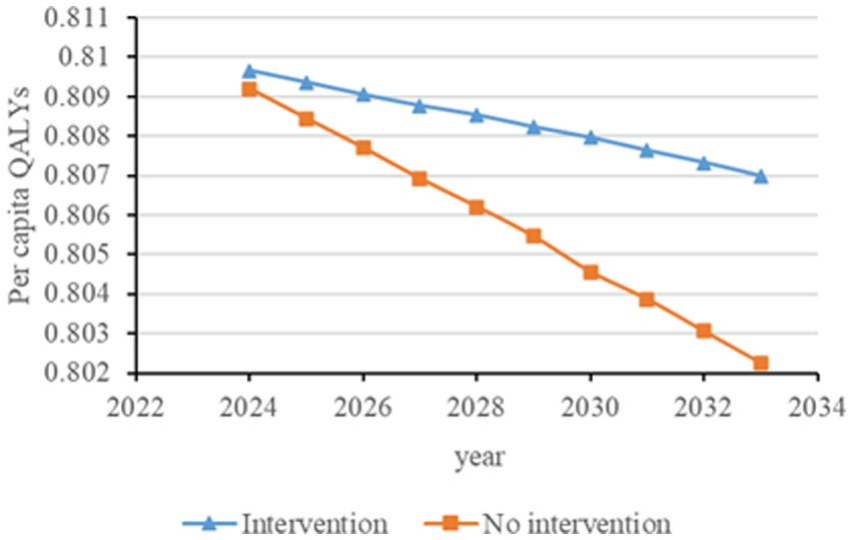
Changes in per capita quality-adjusted life-years before and after screening and intervention for individuals aged 65 years and older. QALYs, quality-adjusted life-years.

**Figure 6:**
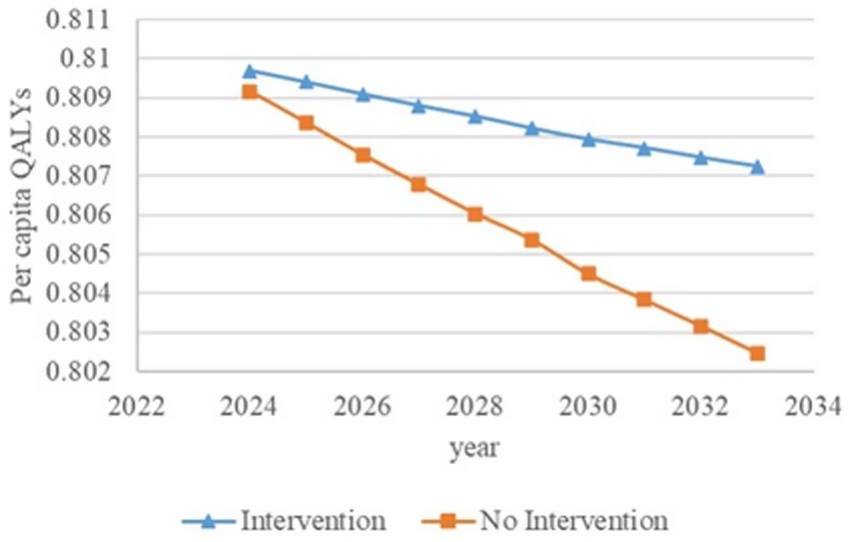
Changes in per capita quality-adjusted life-years before and after screening and intervention for individuals with hypertension, diabetes, and an age of 65 years and older. QALYs, quality-adjusted life-years.

Figures 2–6 illustrate the changes in annual QALYs per capita resulting from screening interventions in different population groups. It can be observed that, over time, the quality of life in both the groups with and without screening interventions decreases annually. However, compared with the group without screening intervention every year, the group with screening intervention consistently exhibited a significantly higher quality of life.

Table 6 shows variations in the economic burden of disease for patients with end-stage renal disease in the hypertension, diabetes, and older adult groups under CKD screening intervention. According to the results, screening intervention effectively reduces the overall economic burden of patients with end-stage renal disease. By 2033, the annual cost savings for the screened and intervened group are predicted to amount to 454 million yuan.

**Table 6:** Changes in the economic burden of end-stage renal disease for hypertension, diabetes, and older adult groups.

Year	Intervention (million yuan)	No intervention (million yuan)	Cost savings (million yuan)
2024	74	199	125
2025	138	360	222
2026	207	516	309
2027	257	643	386
2028	313	757	444
2029	357	875	518
2030	381	963	582
2031	408	1,033	625
2032	445	1,101	656
2033	478	1,148	670
Average	306	760	454

### 3.4 Results of sensitivity analysis

The parameters influencing the simulation analysis included the transition probabilities of CKD, costs, and utility values. These parameters were adjusted, and a single-factor sensitivity analysis was conducted.

**Figure 7:**
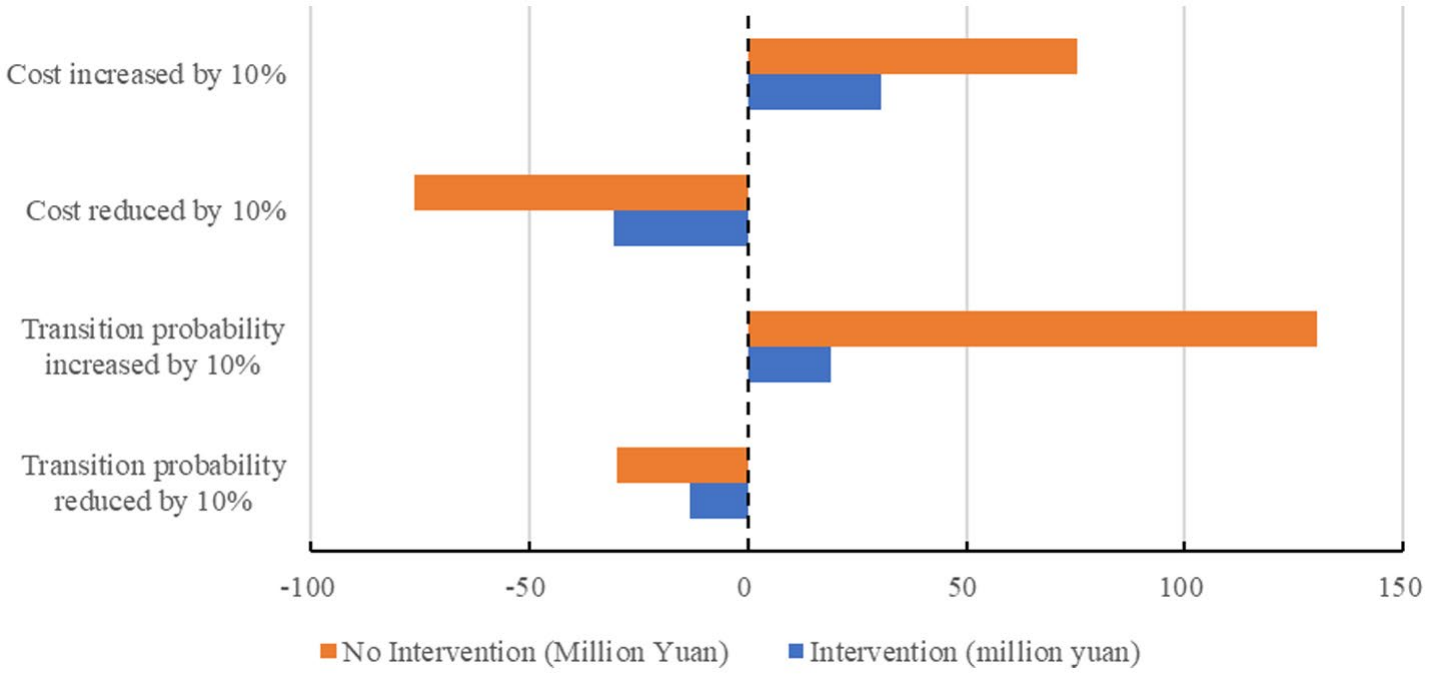
Single-factor sensitivity analysis results of the impact of disease state transition probability and cost changes on the economic burden of diseases (Older Adult, Hypertension, and Diabetes Groups).

Figure 7 illustrates the changes in the annual average disease economic burden of the older adult, hypertension, and diabetes groups when the CKD transition probability and cost increase or decrease by 10%. The single-factor sensitivity analysis results show that cost changes have a greater impact on the economic burden of CKD, and the disease economic burden fluctuates within a certain range. Meanwhile, the economic burden of disease in the intervention group was lower than that in the non-intervention group.

Table 7 illustrates the impact of a 10% increase or decrease in transfer probability and utility value on the per capita QALYs values of the older adult, hypertension, and diabetes cohorts in 2033. Changes in utility values show a greater impact on per capita QALYs.

**Table 7:** Single-factor sensitivity analysis results of the impact of disease state transition probability and utility value changes on QALYs.

Influence factor	Intervention (year)	No intervention (year)
Disease state transition probability reduced by 10%	0.0003	0.0009
Disease state transition probability increased by 10%	−0.0001	−0.0009
Utility reduced by 10%	−0.0807	−0.0803
Utility increased by 10%	0.0784	0.0803

QALYs, quality-adjusted life years.

## 4 Discussion

### 4.1 Early screening intervention for CKD effectively reduces disease burden

First, we found that screening interventions for high-risk groups with CKD can significantly reduce the number of patients with end-stage renal disease. As indicated by the simulation results, the number of patients with end-stage renal disease gradually increases from 2024 to 2033 when considering changes in the scope of screening interventions for various groups. However, the number of patients with end-stage disease subjected to intervention is significantly lower than that without intervention. Expanding the scope of screening intervention for high-risk groups further decreases the number of patients with end-stage renal disease. By 2033, screening intervention for high-risk groups, including individuals with hypertension, diabetes, and an age of 65 years and older, can reduce the number of patients with end-stage disease by 6,250 individuals. This would substantially reduce the disease burden in these groups.

Second, screening interventions for groups at a high risk of CKD effectively improve the quality of life of the population. Our simulation results show that, compared with no screening intervention, screening intervention for groups with hypertension, diabetes, and those aged 65 years and older can enhance the QALYs of the population, reflecting an improvement in their overall quality of life. When analyzing these high-risk groups individually, the greatest increase in QALYs occurs when screening intervention is implemented for individuals aged 65 years and older, with an increase of 0.21 years. This is closely followed by the diabetes group, which shows an increase of 0.20 years, while the hypertension group shows the lowest increase at 0.19 years. This suggests that screening interventions for individuals aged 65 years and older can significantly enhance the overall quality of life among the population.

Third, screening interventions for high-risk groups with CKD effectively reduce the economic burden of end-stage renal disease treatment. End-stage renal disease treatment incurs high costs, imposing a substantial economic burden on both society and families. From the simulation results, it can be observed that screening interventions for high-risk groups can effectively reduce the number of patients with end-stage disease. In 2033, when screening interventions are applied to high-risk groups comprising individuals with hypertension, diabetes, and an age of 65 years or older, the number of patients with end-stage renal disease is only 41.64% of the count without screening interventions. Therefore, implementing screening interventions for all these high-risk groups may annually reduce the end-stage renal disease intervention expenses by 454 million yuan.

### 4.2 Integrated prevention and treatment for chronic diseases provides guidance for effectively utilizing limited healthcare resources to address the high burden of CKD

In the 1970s, Western countries began establishing integrated systems for the prevention and treatment of chronic diseases. Countries such as the United States, the United Kingdom, and Japan adjusted and restructured their healthcare service systems to improve the quality and efficiency of healthcare services, forming integrated healthcare service systems. Research in foreign countries covers the outcomes, management, and quality of integrated healthcare services, the analysis of factors and mechanisms affecting these services, and the evaluations of methods and effectiveness for special groups (14–17). Since 2009, research on integrated prevention and treatment in China has gradually increased, with a significant rise in research papers published after 2015. Research has focused on the construction, modeling, and evaluation of integrated healthcare service systems, as well as on analyzing the construction of integrated models in developed countries and their implications for China. Both domestic and international research results generally support the idea that integrated healthcare service models can reduce hospitalization rates and medical expenses for chronic diseases, improve the health and quality of life of patients, enhance service quality, and reduce diagnosis and treatment time.

In accordance with the hierarchical diagnosis and treatment system, Shanghai has piloted the construction of a comprehensive CKD screening, diagnosis, and treatment system in the Jing’an District. The district has established a three-tier prevention and treatment network that includes early screening for high-risk groups, along with follow-up, diagnosis, treatment, and intervention for patients. These measures align with the concept of integrated prevention and treatment. Judging by the results of this micro-simulation experiment on screening interventions, regular screening of individuals aged 65 years and older, as well as those with hypertension and diabetes, and the provision of timely and effective interventions for identified patients can improve patients’ quality of life and reduce their economic burden. Based on Shanghai’s practices, public health outcomes could be improved by implementing integrated prevention and treatment measures for these high-risk groups. However, in practice, healthcare institutions at all levels must effectively implement their responsibilities and tasks within the diagnosis and treatment system in order to realize the goals of the screening and diagnosis system.

### 4.3 Exploring integrated prevention and treatment of CKD provides practical experience in reducing disease burden

Integrated prevention and treatment refers to a patient-centered approach that integrates the management and provision of various healthcare services, including health promotion, disease prevention, treatment, and end-of-life care. It involves the coordination of various healthcare institutions to provide lifelong continuous services to the population based on their health needs. According to the concept of integrated prevention and treatment, the functions of healthcare institutions at all levels are effectively integrated to prevent and control diseases. From the pilot practice of integrated prevention and treatment of CKD in Shanghai, community-level healthcare institutions mainly engage in early screening of groups at a high risk of CKD, while secondary and tertiary healthcare institutions primarily focus on the treatment of the disease. Therefore, the integrated prevention and treatment process in Shanghai includes both prevention (screening) and treatment (intervention), effectively integrating these aspects of care.

Shanghai has piloted a “27 + 8 + 3” CKD specialty referral mechanism, involving 27 community health service centers, eight district-level hospitals, and three municipal-level hospitals. The city has built up a three-tier prevention and treatment network, with each level of healthcare institution responsible for specific tasks. Early screening of high-risk groups, along with follow-up, diagnosis, treatment, and patient intervention, is carried out effectively as part of the system (18). Results from the pilot program show that the patients’ loss-to-follow-up rate was maintained below 10%, the screening rate for renal damage indicators among high-risk groups reached 70%, the patient filing rate reached 80%, compliance with bidirectional referrals reached 70%, the incidence of end-stage CKD was reduced by 10%, and the incidence of CKD combined with cardiovascular events was reduced by 15%. These results demonstrate the effectiveness of this system’s construction as an integrated prevention and treatment approach for CKD in Shanghai (19).

Moreover, guided by the concept of integrated prevention and treatment, China has gradually established a national Chronic Kidney Disease Management Center (CKDMC) since 2020 (21). Its purpose is to build China’s capacity to prevent and treat CKD and construct a national research network for kidney disease prevention, diagnosis, and treatment. For example, the First Affiliated Hospital of Xi’an Jiaotong University relies on the CKDMC to promote the standardization of the whole-process management, diagnosis, and treatment of CKD throughout the region (20). Both models emphasize the importance of integrated prevention and treatment. Comparatively, Shanghai’s integrated prevention and treatment system has clearer responsibilities and closer connections between institutions within the system, effectively implementing policies. This is the key to Shanghai’s success in achieving integrated prevention and treatment of CKD. However, the effectiveness of prevention and treatment needs to be further improved by enhancing the screening and management capabilities at the community level during the implementation process. Apart from clarifying the responsibilities at each level, building effective collaborative mechanisms among different levels within the integrated prevention and treatment system and strengthening screening and management capabilities at the community level are essential not only for achieving integrated prevention and treatment of CKD, but also the core issues that need to be addressed in the process of system construction. Table 8 lists the core content of the two modes.

**Table 8:** Comparison of integrated prevention and treatment modes for CKD.

Type of mode	Shanghai CKD early detection and treatment system	CKDMC at the first affiliated hospital of Xi’an Jiaotong university
System construction	A three-tier CKD early detection, early prevention, and early diagnosis network consisting of community health centers, district-level hospitals, and municipal-level hospitals	A four-tier network system with “full coverage and tiered” structure, based on the CKDMC
Key initiatives	Community health centers: CKD screening base responsible for initial screening assessment and health education of high-risk groups District-level hospitals: CKD assessment base responsible for categorizing CKD risks in initially screened patients based on eGFR and ACR, and formulating intervention measures. Stable patients after diagnosis are referred to community health service centers. Municipal-level hospitals: CKD treatment base responsible for further specialized examinations and development of treatment and follow-up plans for referred patients with CKD. Stable patients after treatment are referred to district-level hospitals or community health service centers for further treatment and follow-up.	1. Collaborate with other units in the healthcare network of the province to strengthen early screening, early diagnosis, early treatment, specialized treatment, and peri-dialysis management. 2. Establish a CKD difficult and critically ill consultation and remote consultation center to gradually achieve province-wide coverage and standardization of the whole process of CKD management, diagnosis, and treatment. 3. Comprehensively implement the advanced concept of prioritizing prevention in kidney disease, complete early screening, and standardize diagnosis and treatment, with the aim of further improving the prognosis of patients with CKD and delaying disease progression.
Actual effects	Pilot results: Lost-to-follow-up rate within 2 years below 10%, kidney injury indicator screening rate among high-risk groups reaching 70%, patient filing rate reaching 80%, bidirectional referral compliance rate reaching 70%. Achieved the goal of reducing the incidence of end-stage CKD by 10% and reducing the incidence of cardiovascular events in patients with CKD by 15%.	Short establishment time and lack of effective assessment of corresponding implementation effects.

CKD, chronic kidney disease; eGFR, estimated glomerular infiltration rate.

## 5 Conclusion

According to our results, it is recommended to incorporate the concept of integrated prevention and treatment of chronic diseases and continue to explore the construction of the Shanghai CKD screening and prevention system. It is necessary to strengthen collaboration among institutions within the system, effectively implement the key measures of the three-tier CKD prevention and treatment network, and conduct screening and intervention for CKD in individuals with hypertension, diabetes, and an age of 65 years and older. This approach has the potential to effectively reduce the number of patients with end-stage disease, reduce the cost of treatment and intervention, and improve the quality of life of the population.

The single-factor sensitivity analysis results show that the factors affecting the economic burden of CKD are mainly the probability of disease state transition and the cost of disease intervention. In case of a 10% increase or decrease in both, except for the non-intervention group, the change in the probability of disease state transition has a greater impact on the economic burden of disease, whereas the rest are more affected by cost changes. Regarding quality of life, the main factors affecting the annual average QALYs are utility value and probability of disease state transition. When both factors increase or decrease by 10%, changes in utility value have a greater impact on the results.

This study had a few limitations. First, we assumed that the distribution of high-risk groups participating in the screening of CKD is consistent with the distribution of the registered residence population in Shanghai, which may have led to certain errors in the simulation results. Second, the disease state transition probability, mortality, and prevalence of population high-risk factors (hypertension, diabetes) for CKD in the research process are from relevant literature reports or meta-analysis results; therefore, the accuracy of their parameters may also lead to simulation errors to a certain extent. Third, we analyzed the risk of disease onset with and without intervention; therefore, compared with the actual situation, it may, to some extent, expand the screening intervention effect. Fourth, this model assumed full participation in the screening but did not consider the impact of changes in population participation in screening interventions on the results. However, the targeted implementation of prevention and control strategies affects the distribution of screening and treatment intervention behaviors among the affected population, thereby influencing the distribution of screening results and the probability of disease state transition. The probability of disease state transition affects the outcome of disease burden in the population.

## Data Availability

The raw data supporting the conclusions of this article will be made available by the authors, without undue reservation.
